# The Kinase PDK1 Is Essential for B-Cell Receptor Mediated Survival Signaling

**DOI:** 10.1371/journal.pone.0055378

**Published:** 2013-02-05

**Authors:** Sung-Gyoo Park, Meixiao Long, Jung-Ah Kang, Woo-Seok Kim, Cho-Rong Lee, Sin-Hyeog Im, Ian Strickland, Jan Schulze-Luehrmann, Matthew S. Hayden, Sankar Ghosh

**Affiliations:** 1 Department of Microbiology & Immunology, Columbia University, College of Physicians & Surgeons, New York, New York, United States of America; 2 School of Life Sciences and Immune Synapse Research Center, Gwangju Institute of Science and Technology (GIST), Gwangju, Republic of Korea; 3 Department of Dermatology, Columbia University, College of Physicians & Surgeons, New York, New York, United States of America; Maisonneuve-Rosemont Hospital, Canada

## Abstract

Phosphoinositide-dependent kinase 1 (PDK1) plays an important role in integrating the T cell antigen receptor (TCR) and CD28 signals to achieve efficient NF-κB activation. PDK1 is also an important regulator of T cell development, mediating pre-TCR induced proliferation signals. However, the role of PDK1 in B cell antigen receptor (BCR) signaling and B cell development remains largely unknown. In this study we provide genetic evidence supporting the role of PDK1 in B cell survival. We found PDK1 is required for BCR mediated survival in resting B cells, likely through regulation of Foxo activation. PDK1-dependent signaling to NF-κB is not crucial to resting B cell viability. However, PDK1 is necessary for triggering NF-κB during B cell activation and is required for activated B cell survival. Together these studies demonstrate that PDK1 is essential for BCR-induced signal transduction to Foxo and NF-κB and is indispensable for both resting and activated B cell survival.

## Introduction

Phosphoinositide-dependent kinase 1 (PDK1) mediates signaling pathways activated by various cell surface receptors, and is important for cell metabolism, survival, and activation [1]. PDK1 possesses a pleckstrin homology (PH) domain that binds to the second messenger PtdIns(3,4,5)P_3_ [1]. Thus, PDK1 is a well-characterized downstream signaling molecule of phosphoinositide 3-kinase (PI3K) [1], although it has been suggested that PDK1 can phosphorylate substrate without binding to PtdIns(3,4,5)P_3_ [2]. PDK1 may act as a ‘master regulator’ of the protein kinase A/protein kinase G/protein kinase C (AGC) family of kinases [3]. PDK1 was initially identified as a kinase for AKT, and AKT remains the best-characterized PDK1 substrate [3], [4]. AKT activity is regulated by phosphorylation at threonine 308 (T308) by PDK1 [4], [5] and at serine 473 (S473) by mammalian target of rapamycin (mTOR) [6], [7].

Recent studies have established that PDK1 is essential for efficient activation of Protein kinase C θ (PKC-θ) by the PI3K pathway and subsequent assembly of the CARMA1-BCL10-MALT1 (CBM) complex to activate NF-κB during coordinated stimulation of the T cell antigen receptor (TCR) and co-stimulatory receptor CD28 [8]. PDK1 also plays a crucial role in T cell development, as evidenced by the fact that conditional deletion of PDK1 in double-negative (DN) thymocytes blocks T cell development at the DN4 stage by impairing pre-TCR induced proliferation [9]. Deleting PDK1 later, at the double-positive (DP) thymocytes stage, selectively affects CD8SP thymocyte development without altering CD4 single-positive (SP) thymocyte development.

B cells share many features with T cells, most notably antigen receptor triggered and developmentally regulated signaling events (12). Both TCR and B cell antigen receptor (BCR) transmit signals via phosphorylation of immunoreceptor tyrosine-based activation motifs (ITAMs), although in the BCR signaling complex the signal-transducing Igα/Igβ heterodimer only contains two ITAMs, while there are 10 ITAMs in the TCR signaling complex. In BCR signaling, phosphorylated ITAMs mediate recruitment and activation of downstream kinases including SYK and BTK for BCR and Src family kinases (LYN, FYN, and BLK) for pre-BCR [10]. These downstream kinases contribute to PI3K activation. PI3K activation is, in turn, required for activation of NF-κB and AKT and is, therefore, needed for B cell activation [11], [12]. This process is analogous, but not identical, to TCR-induced signaling events. Despite several well-established differences, the ITAM-activated molecules induce serial activation of downstream kinase cascades, including PI3K, JNK, MAPK, PKC, and AKT, and these events are thought to be similar in both T and B cells. Ultimately, this results in activation of several transcription factors (NF-κB, NF-AT, AP-1, *etc*.) necessary for proliferation and survival of both B cells and T cells [13].

The NF-κB pathway affects B cell development and survival [14]. Although it has been reported that PI3K is involved in NF-κB activation downstream of BCR [11], [12], the details of how BCR ligation leads to NF-κB activation through PI3K remain unknown [11], [15]. Similar to TCR signaling, activation of NF-κB downstream of BCR depends upon a PKC isoform, PKCβ [16]. Furthermore, the components of the CBM complex, CARMA1, BCL10 and MALT1, are required for NF-κB activation by both TCR and BCR ligation [16]–[18]. However, despite the clear homology of these signaling pathways, a role for PDK1 in BCR signaling has not been established. In the context of aberrant signaling in lymphoma, PDK1 is essential for NF-κB activation and survival of a diffuse large B cell lymphoma cell line that has a mutation in the BCR proximal signaling adaptor CD79B [19]. Therefore, it is possible that PDK1 is also involved in PI3K induced activation of PKCβ, and subsequent activation of the NF-κB pathway, through assembly and activation of the CBM complex in B cells.

In B cells, the PI3K/NF-κB pathway has been shown to provide signals essential for B cell development and survival. PI3K knockout (p85α) mice show inhibition of B cell development between the pro-B cell and pre-B cell stages, and have impaired immune responses [20], [21]. Deficiency of genes that are involved in the NF-κB pathway has also been found to block B cell development at various stages [22]. The NF-κB pathway regulates expression of many genes that are essential for cell proliferation and survival, including the BCL2 family members BCL2, BCL-xL and Bfl-1/A1; the cell cycle genes E2Fα and cyclin E; and the proliferation-related gene c-Myc [23]. These NF-κB target genes have been found to regulate the survival and proliferation of B cells at various developmental stages. Another PDK1 substrate in B cells, AKT, is known to modulate apoptosis [24], [25] and is involved in T cell development. However, the exact role of PDK1, a major downstream kinase of PI3K, in B cell development remains largely unknown.

In this study, we investigated the role of PDK1 in BCR-induced signal transduction and B cell development using both a mouse with B-cell specific conditional deletion of *Pdk1* and chemical inhibitors of PDK1. We found that PDK1 is essential for B cell development beyond the immature B cell stage in the bone marrow, possibly by helping transduce survival signals triggered by tonic or basal BCR activation. While B cells deficient in PDK1 show a slight reduction in transcription of the NF-κB target genes *Bcl-xl* and *Bcl-2*, they exhibit a profound reduction in the expression of Foxo target genes such as *Aicda*, *Rag1*, *Bcl2l11*, and *p27/Kip,* as seen in the BCR-deleted B cells [26]. Moreover, B cells deficient in PDK1 undergo increased apoptosis after they up-regulate surface IgM at the immature B cell stage. Finally, we demonstrate a requirement for PDK1 in BCR induced activation of NF-κB leading to B cell activation and activated B cell survival. These results establish PDK1 as a regulator of B cell survival by mediating PI3K signaling to both NF-κB and Foxo transcription factors.

## Materials and Methods

### Mice and B Cell Isolation

C.129P2-*Cd19^tm1(cre)Cgn^*/J (CD19-Cre mice) [27] were backcrossed 10 times with C57BL/6 mice. *Pdk1* ^flox/flox^ mice [28] were bred with the CD19-Cre mice, and offspring were bred with *Pdk1* ^flox/flox^ mice to generate CD19-Cre^+^ *Pdk1* ^flox/flox^ and CD19-Cre^+^ *Pdk1* ^+/+^ mice. All mice were backcrossed with C57BL/6 mice for at least 6 generations and all mice were kept in pathogen–free conditions in the animal care facility at Columbia University (New York, New York) and GIST (Gwangju, Korea). All mice experiments were approved by Institutional Animal Care and Use Committee of Columbia University and GIST. Splenic B cells were isolated from normal C57BL/6 mice by negative depletion of non-B cells to a purity of greater than 95%.

### Cell Culture and Antibodies

Isolated primary B cells were cultured in Bruff’s media supplemented with 5% FBS. Anti-PDK1 antibody was purchased from Upstate. Anti-IκBα antibody was purchased from Santa Cruz. Anti-JNK antibody was purchased from Cell signaling. Phosphoantibodies for JNK at T183/Y185 and p38 MAPK at T180/Y182 were purchased from Cell Signaling. Anti-mouse IgM was purchased from Jackson Laboratory. APC-conjugated anti-mouse IgM, FITC-conjugated anti-mouse CD43, PerCP-conjugated anti-mouse CD25, FITC-conjugated anti-mouse CD117, APC-conjugated anti-mouse CD19, PE-conjugated anti-mouse thy1, PE-conjugated-mouse CD4, PerCP-conjugated anti-mouse CD4, APC-conjugated mouse CD8α, PE-conjugated anti-mouse thy1, APC-conjugated anti-mouse B220, and PE-Cy5-conjugated anti-mouse B220 antibodies were purchased from ebiosciences. Anti-PKCβ2 was purchased from Abcam. Anti-HA was purchased from Sigma. Anti-Myc was purchased from Invitrogen.

### In Vivo Reconstitution

Bone marrow cells from bone marrow of CD19-Cre^+^ *Pdk1* ^flox/flox^ and CD19-Cre^+^ *Pdk1* ^+/+^ mice were injected into the veins of the unirradiated Rag1 knockout recipient mice. Recipient mice were sacrificed 6 weeks after transfer. The cell suspension from bone marrow and spleen of recipient mice was stained with fluorophore conjugated antibodies and were analyzed flow cytometry.

### Immunohistochemistry

Spleens were fixed with 4% neutral-buffered formalin and embedded in paraffin. Sections were cut, deparaffinized, and developed in the immunostaining system, using the VECTOR® M.O.M.™ Immunodetection Kit (Vector Laboratory).

### Flow Cytometry

Lymphocytes were isolated from the bone marrow, spleens, and lymph nodes of 6–9 week old mice. Cell viability was assessed by annexin V and 7-amino-actinomycin D staining according to the manufacturer’s protocols (BD-Pharmingen). Stained cells were analyzed on a FACSCalibur.

### Retrovirus Transduction

Phoenix-Eco cells were transfected with pMSCV-Cre-IRES-GFP retroviral vector and recombinant retroviruses were harvested 2 days later after the transfection. Total splenocytes were isolated from wild type mice or *Pdk1* ^flox/flox^ mice and were stimulated with LPS at 10 µg/ml for 24 hours. After stimulation splenocytes were infected with *Cre*-expressing recombinant retroviruses by spin infection. Briefly, splenocytes were pretreated with polybrene for 45 min and the medium was removed. The cells were then incubated with medium containing recombinant retroviruses and polybrene for 45 min and were centrifuged for another 90 min at 2,200 rpm. After centrifugation, virus-containing medium was removed and cells were cultured in fresh medium for the indicated time. Infected cells were analyzed by FACS.

### Immunoprecipitation

Primary B cells were isolated from C57BL/6 mice and the isolated 5×10^7 ^B cells were stimulated with anti-IgM for the indicated time period. The cells were lysed in 1% TritonX-100 lysis buffer containing 20 mM Tris-HCl (pH 7.4), 150 mM NaCl, 1 mM Na_3_VO_4_, and protease inhibitors. Total cell lysates were cleared by centrifugation at 14,000 rpm for 10 min at 4°C. From the cleared lysate, PDK1 protein was immunoprecipitated with anti-PDK1 antibodies. IPs were washed four times with lysis buffer, and analyzed.

## Results

### B Cell Specific *Pdk1* Gene Deletion Dramatically Reduces B Cell Numbers in the Periphery

**Figure 1:**
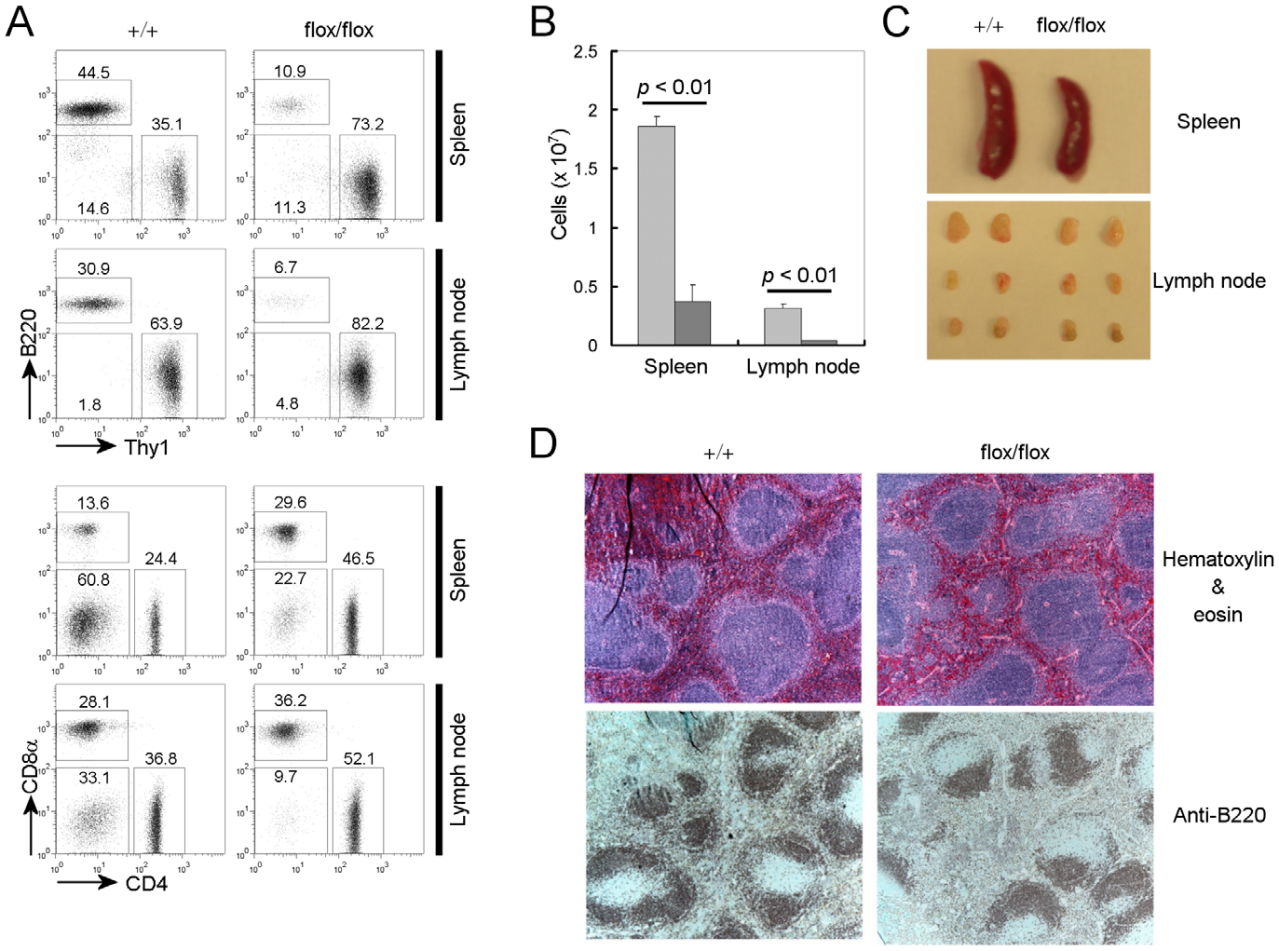
Peripheral B cell numbers are dramatically reduced in B cell specific *Pdk1* knockout mice. (**A**) Flow cytometric analyses were performed with lymphocytes from spleen and lymph nodes of CD19-Cre^+^ *PDK1^+^* ^/+^(+/+) and CD19-Cre^+^ *PDK1* ^flox/flox^ (flox/flox) mice. The FACS plots shown are representative of five different experiments. (**B**) The number of B220^+^ cells in spleen and lymphnode of CD19-Cre^+^ *PDK1^+^* ^/+^ and CD19-Cre^+^ *PDK1* ^flox/flox^ mice (n = 5 mice) presented as mean ± SD. (**C**) Spleen and lymph nodes from CD19-Cre^+^ *PDK1^+/+^* and CD19-Cre^+^ *PDK1* ^flox/flox^ mice. (**D**) Structure of Spleen from CD19-Cre^+^ *PDK1^+/+^* and CD19-Cre^+^ *PDK1* ^flox/flox^ mice. Paraffin-embedded spleen sections from CD19-Cre^+^ *PDK1^+/+^* and CD19-Cre^+^ *PDK1* ^flox/flox^ mice were stained by hematoxylin and eosin (top panel) or anti-B220 (bottom panel).

Based on findings in T cell development and function [8], [9], we hypothesized that PDK1 would also play an important role in B cells. To investigate the role of PDK1 in B cell development, survival and function, we crossed mice in which *Pdk1* was flanked by *lox*P sites (*Pdk1* ^flox/flox^) with mice expressing Cre recombinase under control of the CD19 promoter (CD19-cre mice) to delete *Pdk1* from B cells during bone marrow development. We first analyzed the peripheral B cell population by flow cytometry. As shown in Figure 1, A and B, both the percentage and number of B220 positive peripheral B cells was dramatically reduced in B cell specific *Pdk1* knockout mice, compared with wild type littermate controls. In addition, the size of both the spleen and peripheral lymph nodes was reduced in B cell specific *Pdk1* knockout mice compared to wild type littermate controls (Figure 1C). While B cell numbers are severely reduced by *Pdk1* gene deletion, the reduction of B cells in the spleen does not lead to a gross alteration of splenic structure (Figure 1D). B cells remain in the B cell zone, albeit in dramatically reduced numbers. We also found that the peripheral B cells remaining in PDK1 conditional knockout mice express PDK1 at levels similar to or higher than that of wild type littermate controls. Therefore, remaining peripheral B cells escaped *CD19-Cre* mediated PDK1 deletion.

### PDK1 is Required for B Cell Survival Beginning at the Immature Stage

**Figure 2:**
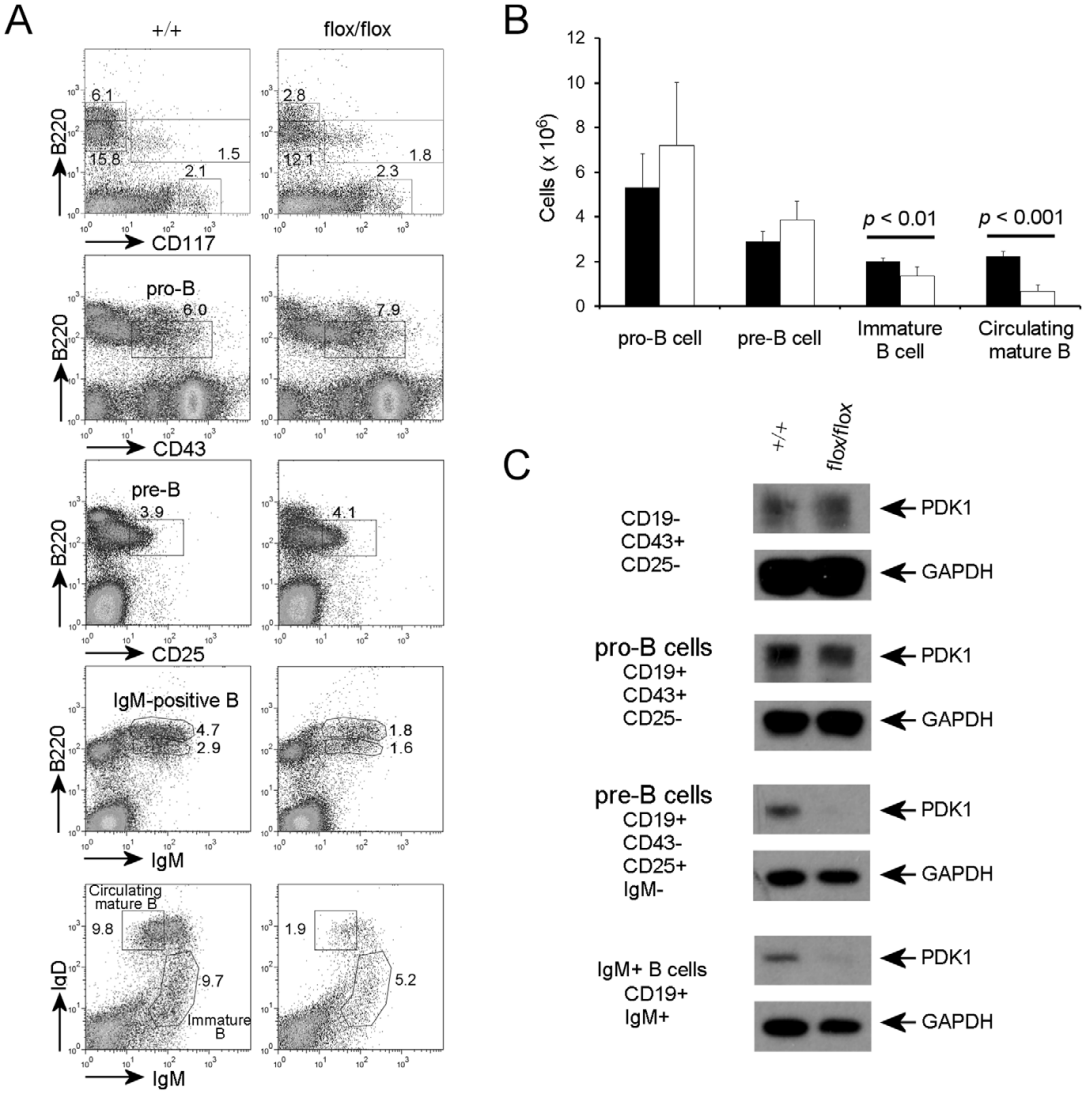
PDK1 deficiency blocks B cell development at the immature B cell stage. (**A**) Flow cytometric analyses of surface markers of B cell lineage development of bone marrow cells from CD19-Cre^+^ *PDK1^+^* ^/+^ and CD19-Cre^+^ *PDK1* ^flox/flox^ mice. The FACS plots shown are representative of five different experiments. (**B**) The number of each B cell lineage cell in bone marrow of CD19-Cre^+^ *PDK1^+^* ^/+^ and CD19-Cre^+^ *PDK1* ^flox/flox^ mice (n = 5 mice) presented as mean ± SD. (**C**) PDK1 deletion was confirmed by immunoblot analysis after sorting of the indicated populations. Total cell lysate of 0.4×10^6^ cells from each population was loaded for this analysis.

Next, we sought to determine the developmental stage at which PDK1 deficient B cells were arrested. Using flow-cytometry, we examined different populations representing key stages of B cell development, including stem cell (B220^−^CD117^+^), pro-B cell (B220^+^CD25^−^CD43^+^), pre-B cell (B220^+^CD25^+^CD43^−^), immature B cell (B220^+^IgM^hi^IgD^lo^), and mature circulating B cell (B220^+^IgM^lo^IgD^hi^) populations (Figure 2A). We did not notice any apparent change in the stem cell or progenitor cell populations in the bone marrow of CD19-Cre^+^ *Pdk1* ^flox/flox^ mice. The total B220^+^ population in the bone marrow of B cell specific *Pdk1* knockout mice was slightly reduced compared with wild type littermate controls (Data not shown). However, the percentage of pro-B cell, and pre-B cells in the bone marrow exhibited no significant differences between B cell specific *Pdk1* knockout mice and littermate controls (Figure 2A).

In contrast to the B cell populations of early developmental stages, immature B cell populations (B220^+^IgM^hi^IgD^lo^) in the bone marrow of PDK1 deficient mice were reduced compared to wild type controls (6.9±1.6% versus 9.5±0.8%). Further analysis using IgM and IgD surface markers showed that the circulating mature B cell population (B220^+^IgM^lo^IgD^hi^) was reduced significantly compared to wild type bone marrow (3.4±0.9 versus 10.5±0.6%). In addition, our data showed that the B cell numbers in *Pdk1* knockout mice are dramatically reduced after the immature B cell stage, i.e. following expression of surface IgM (Figure 2B).

To confirm that these results were not attributable to inefficient deletion of the *Pdk1* gene, B cell populations from each developmental stage were sorted by flow-cytometry and PDK1 expression was determined by western blotting. We loaded cell lysates derived from same number of cells from each population (Figure 2C). Although the total protein recovered from each population differed, likely attributable to differences in cell size, protein recovery was comparable in CD19-Cre^+^ *Pdk1* ^fl/f^ and wild type mice. Using GAPDH as a loading control, we checked the down-regulation of the PDK1 protein level in B cells populations of different developmental stages. In CD19-Cre^+^ *Pdk1* ^fl/f^ mice, PDK1 protein levels were reduced from the pro-B cell (CD19^+^CD25^−^CD43^+^) stage, but the reduction of PDK1 protein level was not dramatic. This might be caused by incomplete deletion of *Pdk1* gene by CD19-Cre in pro-B cell stage. However, at the pre-B cell (CD19^+^CD25^+^CD43^−^) stage, PDK1 protein levels were significantly reduced (Figure 2C). Even though the PDK1 protein level was reduced in cells of the pro-B cell stage from *Pdk1* knockout mice, we could not detect any apparent accumulation of B cells at the specific stage of B cell development. Thus, reduction of the IgM^+^ B cell population in *Pdk1* knockout mice might be caused by loss of the population through apoptosis or proliferative defect.

### Defects in B Cell Development Resulting from *Pdk1* Gene Deletion are B Cell Autonomous

**Figure 3:**
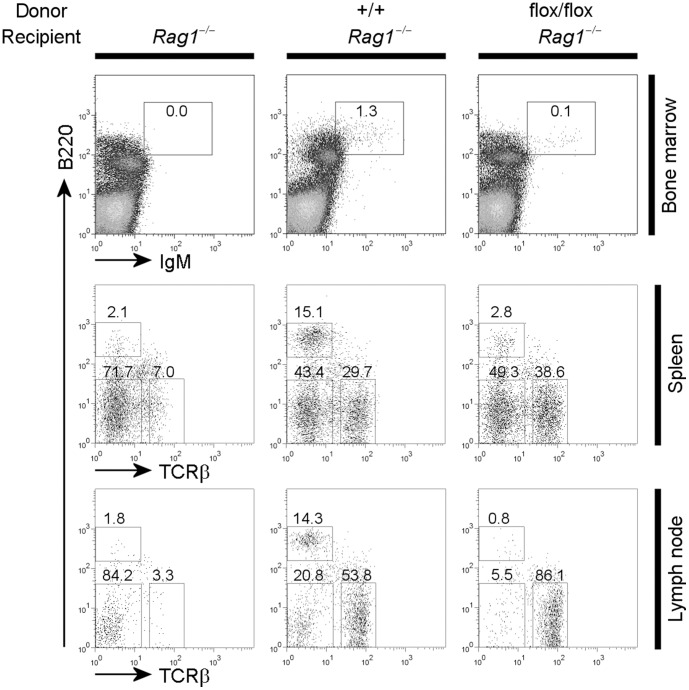
The defect of B cell development in CD19-Cre^+^ *PDK1* ^flox/flox^ mice is caused by B cell intrinsic defects. Bone marrow cells of CD19-Cre^+^ *PDK1^+^* ^/+^ and CD19-Cre^+^ *PDK1* ^flox/flox^ mice were transferred into unirradiated Rag1 deficient recipients. Six weeks after transfer, flow cytometric analyses of B cell surface markers were performed with bone marrow cells, spleen cells and lymph node cells from the recipient mice. This is representative of two independent experiments.

Our observations demonstrate that PDK1 is required for B cell development. However, given that CD19 may be expressed in other cell types, it is possible that *Pdk1* was deleted in cells other than B cells [29]. To rule out the possibility of off-target *Pdk1* deletion affecting B cells development, we performed adoptive transfer experiments by reconstituting non-irradiated Rag1 deficient recipient mice with bone marrow cells from wild type and CD19-Cre^+^ *Pdk1* ^fl/f^ mice. Analysis of reconstituted hosts 6 weeks after transfer demonstrated that recipients that had received wild-type bone marrow reconstituted the pre–B cell and immature B cell compartments (Figure 3). In hosts reconstituted with CD19-Cre^+^ *Pdk1* ^fl/f^ bone marrow, there was an almost complete absence of IgM-positive B cells. In addition to bone marrow, peripheral B cell numbers in recipient mice reconstituted with CD19-Cre^+^ *Pdk1* ^fl/f^ bone marrow were significantly lower compared to mice reconstituted with wild type bone marrow (Figure 3). These findings parallel those obtained by direct analysis of B cell specific *Pdk1* knockout mice and indicate that the observed phenotypes were due to an intrinsic defect of PDK1 deficient B cells.

### PDK1 is Essential for Survival of IgM^+^ B Cells

**Figure 4:**
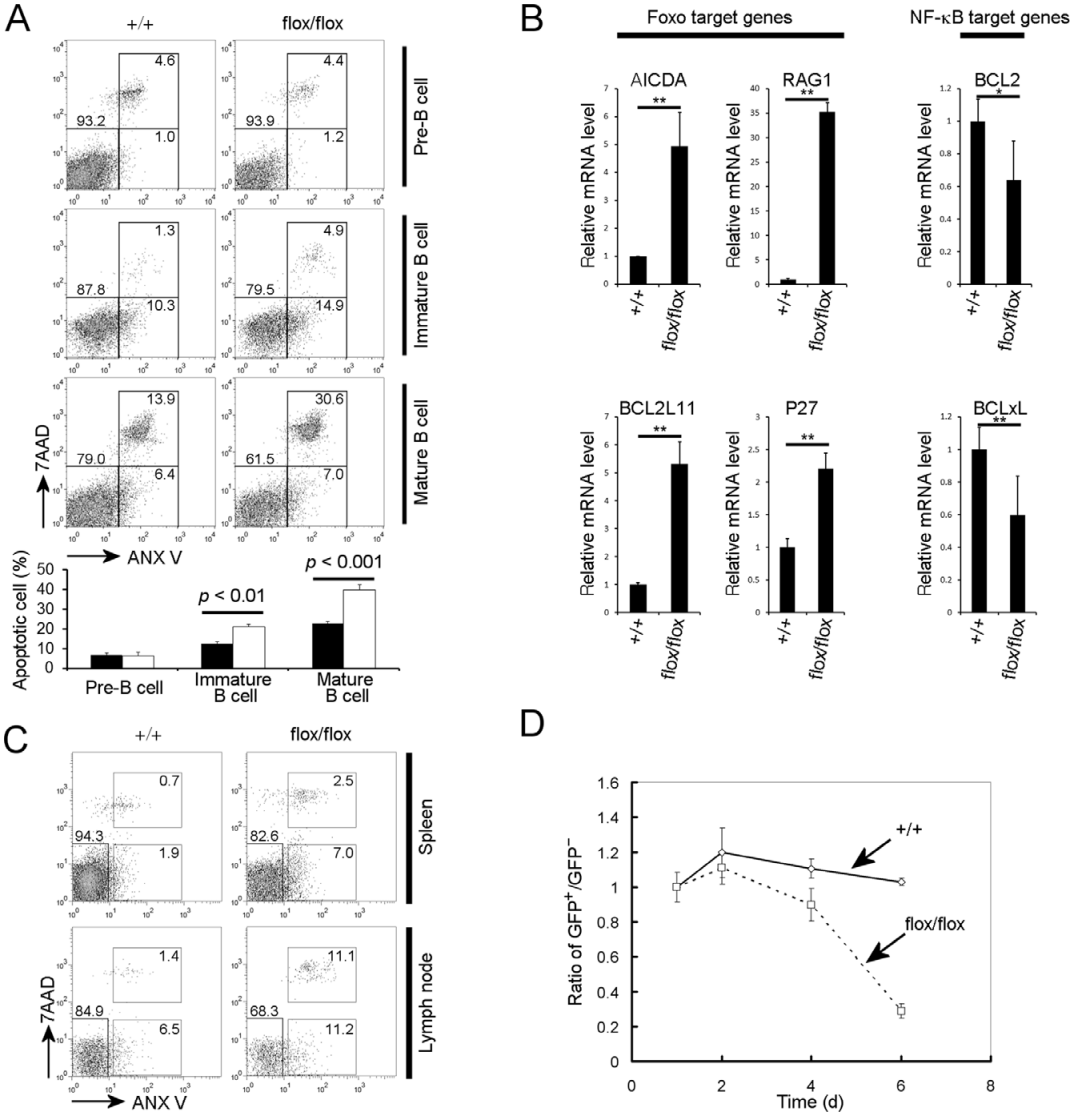
PDK1 is essential for B cell survival after bone marrow immature B cell stage. (**A**) Flow cytometric analyses of apoptosis (as indicated by Annexin V staining) were performed with bone marrow cells of CD19-Cre^+^ *PDK1^+^* ^/+^ and CD19-Cre^+^ *PDK1* ^flox/flox^ mice. The histograms shown were gated as indicated on the right of each plot. Apoptotic percentage of each B cell lineage in bone marrow of CD19-Cre^+^ *PDK1^+^* ^/+^ and CD19-Cre^+^ *PDK1* ^flox/flox^ mice (n = 4 mice) presented as mean ± SD. Numbers indicate the percentage of 7AAD and annexin V–double positive cells (top), annexin V–positive cells (right bottom) or 7AAD–annexin V–negative cells (left bottom). (**B**) Foxo target genes (*Aicda*, *Rag1*, *Bcl2l11* and *p27/Kip*) and NF-κB target genes (*Bcl-2* and *Bcl-xl*) expression levels in IgM-positive bone marrow B cells were analyzed by quantitative RT-PCR analysis as described in the Materials and Methods. Data are presented as mean ± SD (*p<0.05, **p<0.01). (**C**) Flow cytometric analyses of apoptosis markers on B220^+^ B cells were performed with spleen and PLN cells of CD19-Cre^+^ *PDK1^+^* ^/+^ and CD19-Cre^+^ *PDK1* ^flox/flox^ mice. Data are representative of two independent experiments. (**D**) Primary B cells were stimulated with LPS and were then infected with Cre expressing retrovirus (MSCV-Cre IRES GFP). The ratio of GFP-positive to GFP-negative B cell one day after infection was set to 1. Each following day the ratios were checked by flow-cytometry. The experiment was done in triplicate and data are presented as mean ± SD. Data are representative of three independent experiments.

Although the IgM-positive B cell population including immature and mature B cells in bone marrow was significantly reduced by *Pdk1* gene deletion, it was unclear whether this was caused by defective proliferation or excessive cell death of the IgM-positive population. To differentiate between these possibilities, we investigated the cell cycle status at different development stages using DNA content analysis. We found that the percentage of cells in the G2/M stage was considerably greater in B cell populations from CD19-Cre^+^ *Pdk1* ^fl/f^ mice compared to wild type littermate controls (Supplementary Figure 1). This could be due to hyper-proliferation of B cells that escaped *CD19-Cre* mediated deletion, owning to the increased availability of niches due to loss of *Pdk1* deleted cells. We also analyzed the apoptosis status of each population of B cells using annexin-V and 7AAD staining. We found that apoptosis was increased in B cell populations after the IgM-positive immature B cell stage in CD19-Cre^+^ *Pdk1* ^fl/f^ mice, while apoptosis of pre-B cells appeared unchanged (Figure 4A). Consistent with changes in B cell populations in the bone marrow of CD19-Cre^+^ *Pdk1* ^fl/f^ mice (Figure 2A), the apoptotic population is more dramatically increased in the mature B cell population compared with the immature B cell population (Figure 4A).

Foxo is a well-known target of the PI3K/AKT pathway and it is PDK1 that mediates activation of AKT downstream of PI3K. We, therefore, suspected that the defect in survival of IgM^+^ B cells might be due to a failure to inactivate Foxo upon deletion of PDK1. Thus, we tested whether Foxo target genes are differentially regulated after *Pdk1* gene deletion in IgM^+^ B cells. Indeed, *Pdk1* gene deletion in bone marrow IgM^+^ B cell population dramatically increased expression of the Foxo target genes *Aicda*, *Rag1*, *Bcl2l11*, and *p27/Kip* (Figure 4B). However, NF-κB target genes such as *Bcl-2* and *Bcl-xl* were not dramatically down-regulated in bone marrow IgM^+^ B cells after *Pdk1* deletion (Figure 4B).

Peripheral B cell numbers were also dramatically reduced in CD19-Cre^+^ *Pdk1* ^fl/f^ mice, and the remaining peripheral B cells exhibited PDK1 protein levels similar to B cells from wild type mice (data not shown) indicating that these cells evaded *CD19-Cre* mediated deletion of *Pdk1*. In spleen, we found that the mature B cell population is more severely decreased similar to the bone marrow of CD19-Cre^+^ *Pdk1* ^fl/f^ mice and CD19^+^, but IgM^-^ B cell population is increased (Supplemental Figure 2). In both the spleen and peripheral lymph nodes, apoptosis of B cell populations was increased in CD19-Cre^+^ *Pdk1* ^fl/f^ mice (Figure 4C). It has been reported that the deletion process mediated by *CD19-Cre* continues after B cells travel from the bone marrow to the periphery [27]. Therefore, it is likely that a significant proportion of peripheral B cells from CD19-Cre^+^ *Pdk1* ^fl/f^ mice eventually undergo *CD19-Cre* mediated deletion of the *Pdk1* gene, and this de-novo deletion of *Pdk1* in peripheral B cells could result in apoptosis. Consistent with this hypothesis, induction of *Cre* gene expression in mature B cells using recombinant retrovirus infection dramatically reduced B cell survival (Figure 4D).

### PDK1 is Essential for B Cell Receptor-mediated B Cell Activation

As PDK1 is required for B cell survival after the bone marrow immature B cell stage, i.e. after B cells express surface BCR (IgM) that is essential for B cell survival and subsequent maintenance, we speculated that PDK1 is required for BCR mediated survival of B cells. Therefore, we further investigated the role of PDK1 in BCR induced signal transduction. Although the best way to study this would have been to use the *Pdk1* deficient B cells, rapid apoptosis of B-cells lacking *Pdk1* required the use of an alternate approach.

Inducible translocation of the BCR and associated signaling molecules to lipid rafts is essential for efficient BCR triggered signaling and activation of B cells. Therefore, we first tested whether PDK1 is localized to the lipid rafts during BCR activation. We prepared lipid raft fractions following anti-IgM or control IgG treatment, and PDK1 localization to lipid rafts was determined using immunobloting analysis. It is known that IKK is also localized to the lipid raft fraction upon BCR signaling [16], and therefore we used IKK as a marker for BCR-induced lipid raft localization. We consistently detected IKK enriched in the lipid raft fraction upon stimulation. We also observed that PDK1 was enriched in the lipid raft fraction of splenic B cells following anti-IgM treatment (Supplemental Figure 3). Therefore these data indicate that PDK1 is recruited to lipid rafts after BCR ligation and is possibly involved in BCR signaling.

**Figure 5:**
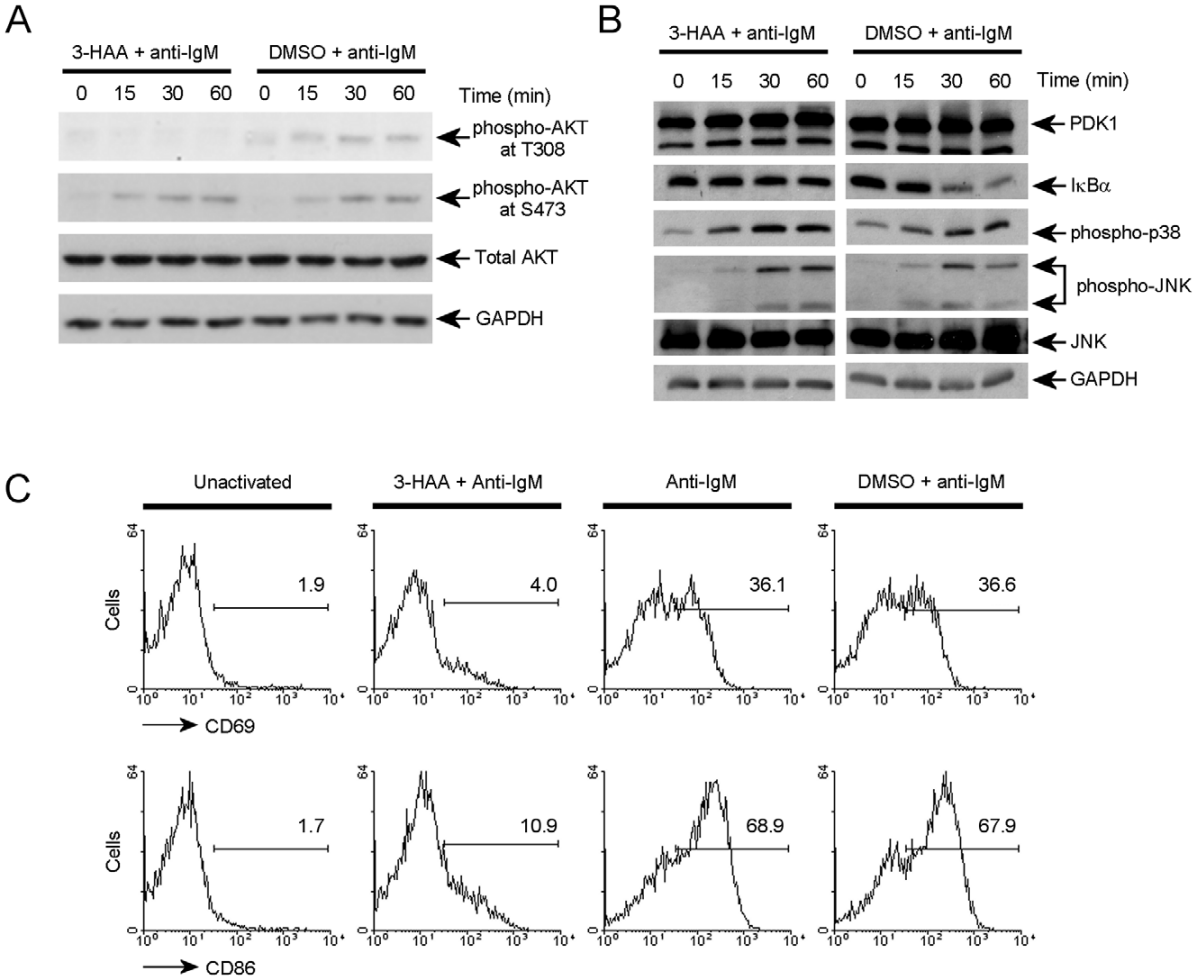
PDK1 is essential for B cell activation. (**A**) Phosphorylation of AKT at T308 and S473 in primary B cells stimulated with AffiniPure F(ab′)2 fragment goat anti-Mouse IgM and with or without 3-HAA were analyzed by immunoblot anlaysis. (**B**) Phosphorylation of JNK and p38 MAPK and IκBα degradation in primary B cells stimulated with anti-IgM antibody and with or without 3-HAA were analyzed by immunobloting analysis. (**C**) Flow cytometric analyses of B cell activation markers (CD69 and CD86) were performed with primary B cells stimulated with anti-IgM antibody for 24 hours and with or without 3-HAA. Numbers indicate the percentage of CD69 or CD86 positive cells. DMSO was used for the vehicle control. (**A**), (**B**) and (**C**) are representative of three independent experiments.

The chemical compound 3-hydroxyanthranilic acid (3-HAA) has been reported to act as a specific inhibitor of PDK1 kinase activity [30]. Therefore, to circumvent our inability to obtain viable PDK1 deficient B cells for biochemical analysis, we used 3-HAA treated B cells to test the role of PDK1 in BCR signal transduction. Inhibition of PDK1 kinase activity was confirmed by defective phosphorylation of the T308 site on AKT after BCR ligation (Figure 5A), a process that is widely accepted to be mediated by PDK1 [3], [4]. On the other hand, treatment of B cells with 3-HAA did not affect BCR-induced phosphorylation of AKT S473 (Figure 5A), an event that is believed to be carried out by the mTOR pathway [6]. Therefore, these results demonstrated that 3-HAA specifically inhibits PDK1 kinase activity. We then tested the effect of 3-HAA on activation of the NF-κB pathway, as indicated by IκBα degradation, in B-cells treated with anti-IgM. While IκB degradation was clearly inhibited (Figure 5B), the two other major downstream signaling pathways induced by BCR ligation, the JNK and p38 MAPK pathways, were not affected by 3-HAA treatment. This specific inhibition of the NF-κB pathway following BCR engagement indicates that 3-HAA treatment does not induce gross abnormalities in B cell signaling, and suggests the impairment of the NF-κB pathway is caused specifically by inhibition of PDK1. It has been reported that 3-HAA treatment of T cells only affects TCR-mediated NF-κB activation and AKT phosphorylation at T308 [30], similar to the results obtained here in B cells.

BCR-antigen engagement activates B cells and this activation induces up-regulations of surface activation markers including CD69 and CD86, which are involved in lymphocyte attachment and antigen presentation, respectively. Our data shows that the inactivation of PDK1 abolished the up-regulation of surface activation markers (Figure 5C), consistent with an essential role for PDK1 in BCR induced NF-κB activation. Together with the failure of survival of B cells lacking PDK1, these data indicate that PDK1 is an important mediator of BCR-mediated B cell activation.

### PDK1 is Required for NF-κB Activation and NF-κB-mediated B Cell Survival

**Figure 6:**
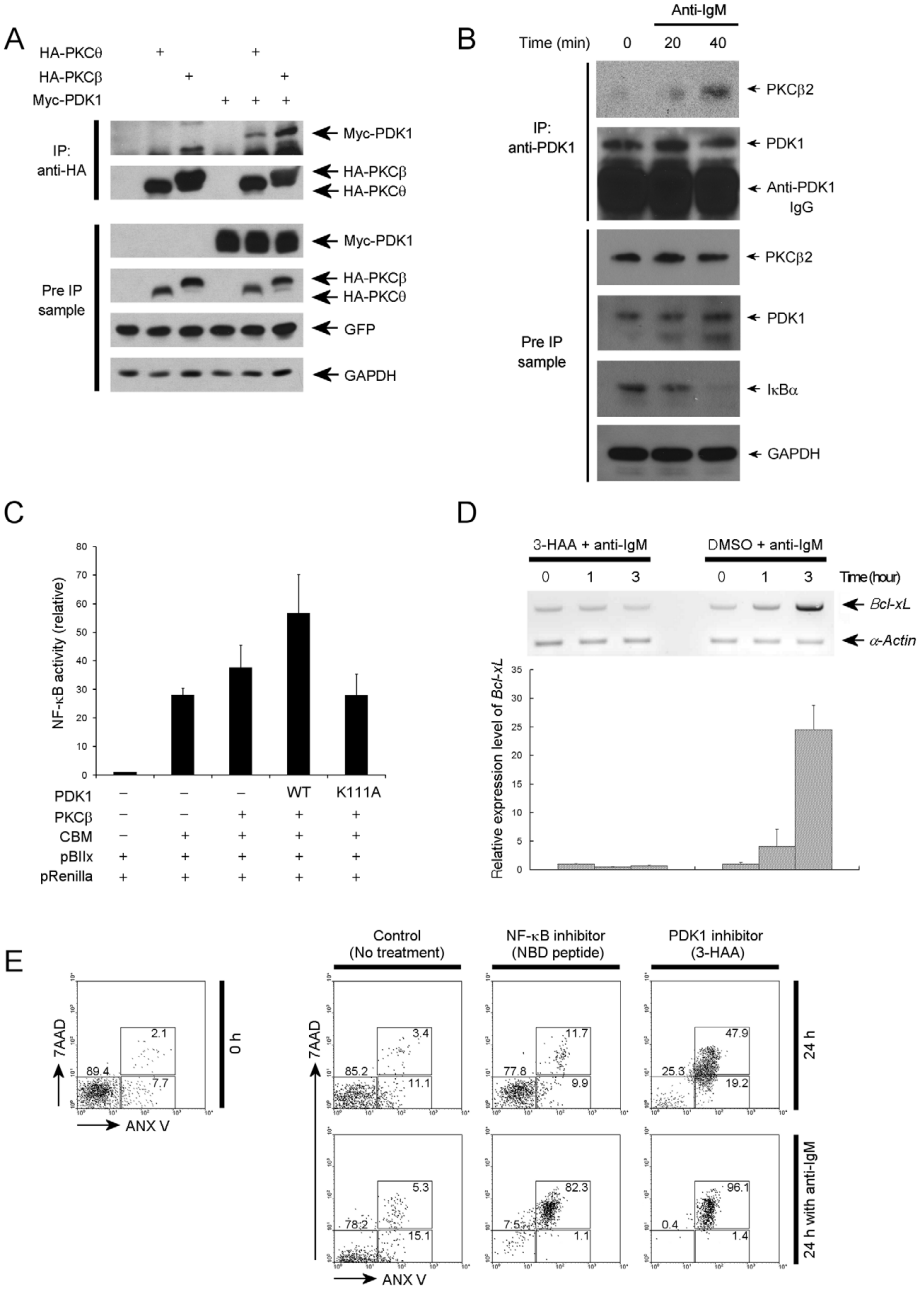
3-HAA (PDK1 inhibitor) inhibits B cell receptor-meditated NF-κB activation. (**A**) HEK293 cell were transfected with plasmids for expression of Myc-PDK1 and HA-PKCβ or HA-PKCθ, the cell lysates were then analyzed for the binding between PDK1 and PKCβ or PKCθ by immunoprecipitation and immunoblotting. In the construct for expression of HA-PKCβ or HA-PKCθ, internal ribosome entry site (IRES) sequence and GFP open reading frame (ORF) is integrated after the PKC ORF. Thus, GFP expression level was used for internal control. (**B**) The B cells stimulated with anti-IgM were analyzed for the binding between PDK1 and PKCβ by immunoprecipitation and immunoblotting. (**C**) Luciferase assay with reporter plasmids containing NF-κB binding sites. Expression plasmids of PDK1, PKCβ, or CARMA1-Bcl10-Malt1 (CBM) were cotransfected as indicated. Data are presented as mean ± SD. (**D**) *Bcl-xl* gene expression was analyzed through quantitative RT-PCR analysis. Data are presented as mean ± SD. (**E**) Flow cytometric analyses of apoptosis markers were performed with primary B cells stimulated with or without anti-IgM antibody and with 3-HAA, NF-κB inhibitor peptide (NBD peptide) or DMSO. Numbers indicate the percentage of 7AAD and annexin V–double positive cells (top), annexin V–positive cells (right bottom) or 7AAD–annexin V–negative cells (left bottom). (**A**), (**B**), (**C**), (**D**) and (**E**) were representative of two to three experiments.

We next investigated how PDK1 functions in the NF-κB activation pathway in B-cells. Previous studies have indicated that NF-κB activation can be induced by BCR ligation via the PKCβ-CARMA1 cascade [17], [18]. We have previously reported that PDK1 is essential for optimal TCR/CD28 induced NF-κB activation through the PDK1-PKCθ-CARMA1 pathway [8]. Therefore, we wanted to test whether PDK1 might also interact with PKCβ, which is the PKC isoform in B cells used to activate the NF-κB pathway. Using co-immunoprecipitation, we found that PDK1 binds to PKCβ, when both proteins are over expressed in HEK293 cells (Figure 6A). In addition, PKCβ, binds to PDK1 in primary B cells after stimulation with anti-IgM (Figure 6B). Moreover, we found that PDK1 and PKCβ, enhanced CBM-mediated NF-κB activation, but kinase inactive PDK1 did not (Figure 6C). Therefore, NF-κB activation during BCR signaling can be mediated by PDK1-PKCβ, activation pathway.

Therefore, in BCR induced signaling, PDK1 is specifically involved in activation of AKT and the NF-κB pathway, both of which are known to be involved in cell survival via regulation of the expression and activity of anti-apoptotic BCL2 family members. As mentioned before, BCL-xL plays an important role in survival of B cells and is induced upon BCR ligation. We found that up-regulation of BCL-xL induced by BCR activation can also be blocked by 3-HAA treatment (Figure 6D). In addition, we found that NF-κB activation is directly linked with activated B cell survival. Treatment with NF-κB inhibitor peptide affects activated B cell survival while the peptide did not affect resting B cell survival (Figure 6E). However, 3-HAA treatment severely affected both resting and activated B cell survival (Figure 6E). These results suggest that PDK1 contributes to survival of B cells stimulated through the BCR by mediating activation of NF-κB and AKT pathways and that PDK1 is essential for survival of resting B cells primarily by mediating activation of AKT.

## Discussion

In T cells, PDK1 has been shown to be a crucial intermediate kinase in the CD28-PI3K pathway, responsible for integrating the TCR and CD28 signaling pathways and allowing their synergistic activation of NF-κB [8]. Engagement of the TCR complex leads to the recruitment of CD28 to the cSMAC region and consequent interaction of CD28 with its ligand, B7. Activated TCR signaling complexes recruit PKCθ, whereas CD28, through PI3K-generated PtdIns(3,4,5)P_3_, recruits and activates PDK1. PDK1 not only interacts with and activates PKCθ, but also interacts with CARMA1, thereby allowing PKCθ to phosphorylate CARMA1, a crucial step in the subsequent assembly of the CBM complex and activation of the NF-κB pathway. Other TCR-CD28–mediated signals, including Jnk, p38 and NFAT pathways, are not affected by PDK1.

BCR signaling resembles TCR mediated signaling in many ways. Cross-linking of the BCR also leads to phosphorylation of ITAMs, those of Igα and Igβ, and activation of proximal signaling molecules including Syk and PI3K. These proximal signaling molecules, in turn, activate downstream signaling pathways including PKCβ, JNK, p38 MAPK, NF-κB, and AKT pathways. In this report, to understand the role of PDK1 in BCR induced signal transduction, we tried to achieve B cell specific deletion of *Pdk1* in *Pdk1* ^flox/flox^ mice using CD19-Cre; however, extensive loss of B cells beginning in the immature B cell stage precluded purification of PDK1 deficient mature B cells for further studies. Therefore, we used a chemical inhibitor that specifically blocks PDK1 activity to examine the role of PDK1 in BCR-induced signaling pathways. We found that, upon BCR stimulation, PDK1 is relocated to lipid rafts and is involved in activation of the NF-κB pathway, but not in the activation of JNK and p38 MAPK pathways. We also found that PDK1 can bind to PKCβ in activated B cells. Our data suggest that upon BCR ligation, PDK1 participates in activation of PKCβ. We speculate that these events lead to activation of NF-κB pathway, in a manner similar to what occurs in T cells following TCR ligation. We also found that PDK1 is involved in BCR-induced activation of the AKT pathway through phosphorylation of T308 of AKT, while the other major phosphorylation site (S473) of AKT is not impacted by the inhibition of PDK1. The T308 site is located on the activation loop of AKT, and the S473 site is located on the hydrophobic motif. It has been reported that the T308 site is regulated by PDK1 [3], [4], while the S473 site is regulated by mTOR-rictor [6]. Therefore, our data suggests that PDK1 plays a crucial role in mediating antigen receptor induced signal transduction in B cells in a manner that is similar to the role of PDK1 in T cells.

In this report, we provide convincing evidence that PDK1 is essential for B cell survival. However we could not clearly define the effect of PDK1 on early B cell development. The timing of the cre-induced *Pdk1* gene deletion in this model did not allow us to assess the role of PDK1 in pre-B cell differentiation. Complete deletion of PDK1 leads to excessive apoptosis and loss of B cells beginning at the immature B cell stage, following up-regulation of surface IgM. Interestingly, although PDK1 is the downstream target of PI3K, PDK1 deficiency, as assessed by PDK1 protein levels in sorted cells, does not appear to affect pre-B cell survival.

We found that the remaining peripheral B cells in CD19-Cre^+^ *Pdk1* ^flox/flox^ mice had escaped *Pdk1* deletion. Nevertheless, CD19-Cre^+^ *Pdk1* ^flox/flox^ peripheral B cells exhibited dramatically increased apoptosis. It seems likely that continuous peripheral *CD19-Cre*-mediated deletion [27] of the *Pdk1* gene in escaped B cells was the cause of the observed increase in apoptosis. Consistent with this hypothesis, we found that deleting *Pdk1* by retrovirus-mediated *Cre* expression also reduced survival in peripheral B cells. These results further support the notion that PDK1 is essential for B cell survival beyond the bone marrow immature stage.

Basal BCR induced signaling, triggered either through an antigen-dependent positive selection process, or through an antigen independent “tonic” signaling process, is crucial for B cell development and survival beyond the immature stage. Recently it has been shown that this survival signal is mediated by the PI3K pathway, largely through regulation of Foxo transcription factors and, in part, through NF-κB [26]. We found that B cell survival becomes dependent on PDK1 after B cells enter the immature B cell stage and up-regulate their surface IgM/BCR complex. In the IgM^+^ B cell, the NF-κB target gene expression is slightly down-regulated by *Pdk1* deletion which suggest that PDK1 is involved in basal BCR-mediated NF-κB activation. However, even though PKCβ and CARMA1 are involved in BCR-mediated NF-κB activation during the B cell activation, the PKCβ [31] and CARMA1 [32] knockout mice show normal survival of resting B cells. Recent data suggested that the “tonic” signaling that is required for resting B cell survival is mediated by regulation of Foxo, but not by NF-κB activation [26]. Consistent with these data we found that Foxo target gene expression was increased in IgM^+^ B cells. Furthermore, NF-κB inhibition had little effect on resting B cell survival. Therefore, PDK1 mediates resting B cell survival through its well known function as a key signaling intermediate in PI3K/AKT signaling. In contrast to the situation in resting B cells, NF-κB blockade dramatically reduced the survival of activated B cells. These data suggests that in these cells, activation of NF-κB may also be an important function of PDK1. Such a model is entirely consistent with previous data demonstrating that deletion of either PKCβ and CARMA1, essential components of the signaling pathway from BCR to NF-κB, only affect the activated B cell survival. Therefore, while PDK1-mediated NF-κB activation is required for survival of activated B cell (strong BCR activation), PDK1-mediated regulation of Foxo could be required for survival of resting B cell (weak BCR activation). The contribution of Foxo to PDK1-mediated survival signals in activated B cells is unclear, but it can be surmised from previous studies that proliferation following BCR activation will also be dependent on PDK1-induced regulation of Foxo [33]. In summary, our results suggest that PDK1 plays a critical role in B-cells, by helping transduce BCR signals that are essential for the regulation of both NF-κB and Foxo transcription factors and thus PDK1 is required for the survival of resting and activated B cells.

## Supporting Information

Figure S1 **Flow cytometric analyses of the cell cycle (7AAD staining) were performed with bone marrow cells of** ***CD19-Cre*** **/** ***PDK1^+^*** ^**/+**^ **and** ***CD19-Cre*** **/** ***PDK1*** **^flox/flox^ mice.** The histograms shown were gated for IgM^+^B220^+^ cells. The area parameter histogram was used to determine the percentage of cells in sub-G1/G0 (first area), G1/G0 (second area), S (third area), and G2/M (fourth area) phases.
(AI) Click here for additional data file.

Figure S2 **Flow cytometric analyses of surface markers of B cells in spleen cells from CD19-Cre^+^** ***PDK1^+^*** ^**/+**^ **and CD19-Cre^+^** ***PDK1*** **^flox/flox^ mice.** The bottom dot plots shown were after gating by CD19^+^.
(AI) Click here for additional data file.

Figure S3 **Redistribution of PDK1 to lipid raft after BCR stimulation.** Primary B cells were stimulated with anti-IgM antibody or IgG control and were then lysed with 1% Triton X-100 (Sigma) and subjected to sucrose density gradient centrifugation to isolate lipid rafts. The distribution of PDK1 in lipid and non-raft fraction was assessed by immunoblotting.
(AI) Click here for additional data file.
